# A neuroscientific evaluation of driver rehabilitation: Functional neuroimaging demonstrates the effectiveness of empathy induction in altering brain responses during social information processing

**DOI:** 10.1371/journal.pone.0232222

**Published:** 2020-04-29

**Authors:** Daniel J. Shaw, Kristína Czekóová, Beáta Špiláková, Miguel Salazar, Pavel Řezáč, Veronika Kurečková, Petr Zámečník, Milan Brázdil

**Affiliations:** 1 Behavioural and Social Neuroscience Research Group, Central European Institute of Technology (CEITEC), Masaryk University, Brno, Czech Republic; 2 Department of Psychology, School of Life and Health Sciences, Aston University, Birmingham, United Kingdom; 3 Transport Research Centre (CDV), Brno, Czech Republic; Tongii University, CHINA

## Abstract

An alarming number of traffic-related deaths occur each year on European roads alone. Figures reveal that the vast majority of road-traffic accidents are caused by drivers themselves, and so further improvements in road safety require developments in driver training and rehabilitation. This study evaluated a novel approach to driver rehabilitation–specifically, empathy induction as a means of changing attitudes towards risky driving. To assess the effectiveness of this method, the present study employed functional magnetic resonance imaging (fMRI) to compare brain function before and after a short program of empathy induction in 27 drivers whose licenses had been revoked after serious traffic offences (rehabilitated drivers [RDs]). In an extension of our previous research, we first assessed whether neural responses to empathy-eliciting social stimuli changed in these RDs. In order to isolate the neurophysiological effects of empathy induction from any other potential influences, we compared these RDs to a sample of 27 age-, handedness- and driving experience-matched control drivers (CDs) who had no exposure to the program. We then performed dual-fMRI “hyperscanning” to evaluate whether empathy induction changed brain responses during real-world social interactions among drivers; namely, during co-operative and/or competitive exchanges. Our data reveal that RDs exhibited weaker brain responses to socio-emotional stimuli compared with CDs prior to the program, but this difference was reversed after empathy induction. Moreover, we observed differences between pre- and post-program assessments in patterns of brain responses in RDs elicited during competitive social exchanges, which we interpret to reflect a change in their proclivity to react to the perceived wrong-doing of other road users. Together, these findings suggest that empathy induction is an effective form of driver rehabilitation, and the utility of neuroscientific techniques for evaluating and improving rehabilitation programs.

## 1. Introduction

The World Health Organisation (WHO) has estimated that the annual number of road traffic deaths is around 1.35 million, becoming the leading killer of people aged 5–29 years. On EU roads alone, there are more than 25,000 fatalities and 135,000 serious injuries every year, incurring annual socio-economic costs of around €120 billion. The European Commission for Mobility and Transport responded to this with an action plan to half the number of road deaths by 2020. In 2017, however, it reported a reduction of only 20%. Although this decrease demonstrates marked improvement in road safety, it also highlights the urgent need for further progress. Importantly, figures for specific accidents show that the vast majority are caused by drivers themselves; of all road fatalities in Europe, approximately 30% involve speeding and an additional 25% are alcohol-related. In this light, improving road safety requires the development of more effective techniques for driver training and rehabilitation.

In combination with a penalty point system, many countries employ driver rehabilitation as a means of preventing recidivism for serious driving offences associated most frequently with road deaths (e.g., speeding, driving under the influence of alcohol). Several EU projects aimed at improving road safety have recommended that rehabilitation programs address the beliefs and attitudes that influence driving-related behaviours and decisions; projects GADGET [1], ANDREA [2] and SUPREME [3], for example, have all promoted a change in attitudes around risky driving as means of achieving behavioural adaptation. Evaluating the effectiveness of driver-rehabilitation programs has proven to be problematic, however, impeding their improvement and the development of new techniques. Using recidivism rates or accident figures, some studies report the positive effects of therapeutic rehabilitation [4,5] while others report no additive effect over basic skills training [6]. These discrepancies likely reflect the inadequacy of such metrics for evaluative criteria [6]; the accuracy of these data relies entirely on the variable practices of authorities in imposing penalties. Clearly, then, alternative methods for objective evaluation are required if we are to improve existing driver training and rehabilitation [7].

In a previous study, we employed neuroimaging methods to evaluate the effectiveness of a national traffic-safety campaign launched in Czechia–*Nemyslíš*, *zaplatíš* (“If you don’t think, you will pay”). With 62 fatalities per million inhabitants recorded in 2018, representing one of the lowest reductions in road fatalities among EU member states, the risk of dying in a road accident in Czechia is approximately twice as high as it is in the UK (European Commission, 2018). Again, various statistics converge to show that most road accidents in Czechia are caused by drivers; 39% of all fatalities recorded in 2018 were attributed to excessive speed and 11% to alcohol-related crashes. In an effort to address this through attitudinal change, this campaign involved short videos broadcasted widely that were designed to elicit empathy and compassion towards victims of reckless driving behaviour. With functional magnetic resonance imaging (fMRI), we were able demonstrate that the campaign videos did indeed engage brain systems implicated in these socio-emotional processes [8]. Furthermore, we also revealed that a sample of drivers whose licences had been revoked following serious traffic offences exhibited reduced neural responses to these videos within the same brain systems compared to drivers with no traffic violations [9]. The ability of fMRI to detect such differences in brain responses during social information processing led us to question whether this neuroscientific technique can also be used to measure the effectiveness of driver rehabilitation at the neural level.

To investigate this, the present study acquired fMRI data from a sample of drivers before and after they completed a state-sponsored rehabilitation program having had their licence revoked due to serious traffic offences. Following EU guidelines, this driver-rehabilitation program provides both educational and therapeutic intervention as a means of changing attitudes surrounding risky driving behaviour. More specifically, this particular program employs techniques of empathy induction as means of changing attitudes towards risky driving; rehabilitation requires drivers to reflect on the victims of road traffic accidents as a means of eliciting the socio-emotional processes behind compassion. Driven by our earlier findings, we first evaluated the effectiveness of this approach to driver rehabilitation by imaging drivers’ brains while they watched videos from the campaign before and after empathy induction. This allowed us to examine whether empathy induction is capable of changing the neural responses to these stimuli in drivers undergoing the program. Importantly, to isolate any effects of empathy induction from potentially confounding practice or familiarity effects, we also scanned the brains of drivers who did not participate in the program. This group provided a baseline against which the rehabilitated drivers were compared.

In an extension of our previous finding, we also investigated how altered socio-emotional brain responses in these drivers might manifest in their interactions with other road users. To achieve this, we also imaged pairs of drivers’ brains simultaneously whilst they interacted with one another in co-operative or competitive exchanges. There is a growing awareness that such “hyperscanning” paradigms are essential if we are to understand how the brain processes and responds to social information in real-world situations [10,11]. Indeed, using the hyperscanning method we have begun to elucidate the brain systems that respond to the behaviour of others during social exchanges and produce reciprocal reactions [12,13]. Moreover, an increasing number of hyperscanning studies report that the brain signals of two or more interacting individuals become coupled, or aligned, revealing an indirect chain of inter-brain events that underlie reciprocal behaviour during social exchange [14]; for a review see [15]. By performing fMRI hyperscanning before and after drivers completed this empathy induction program, we assessed whether advanced analytical techniques for these data are sensitive enough to detect post-program changes in drivers’ brain responses during social information processing.

## 2. Methods

### 2.1 Participants

This study involved two samples of drivers: The first comprised 36 males who were required to complete rehabilitation between May 2016 and November 2017 having had their driving licence revoked following one or more serious traffic violations. The second consisted of 36 male drivers who had no record of driving-related convictions, nor any involvement in the program. Herein we refer to these two groups as Rehabilitated Drivers (RDs) and Control Drivers (CDs), respectively. Individuals from the RD group were paired with a driver from the CD group matched on age, self-evaluated handedness and driving experience, the last of these indexed by the length of time participants had held a driving licence at the point of pre-program assessment. The CD group provided a reference against which the brain and behavioural data of the RD group were compared to measure the potential effects of empathy induction in isolation of any general practice and/or habituation effects. Specific RD-CD pairings were maintained throughout the study.

A high rate of attrition between the two assessments meant that only 27 RDs completed the entire experimental paradigm. The paper presents the data from this sub-set of RDs and their matched CDs (mean age = 34.90 [standard deviation ± 10.08] years; mean driving experience = 17.69 [± 9.47] years). The mean difference in age and driving experience between paired drivers was 0.87 (±0.81) and 1.44 (±5.73) years, respectively. All participants had normal or corrected-normal vision, all reported no history of psychology or psychiatric disorder, and 23 pairs comprised individuals reporting themselves to be predominantly right handed. The study was approved by the Research Ethics Committee of Masaryk University, and all participants provided written informed consent prior to the experimental procedure.

### 2.2. Procedure

All individuals in the RD group attended a rehabilitation program delivered by the Transport Research Centre (www.cdv.cz). This program comprised five weekly group meetings (8–15 participants), each delivered by trained psychologists and/or therapists over approximately four hours. Meetings combine both educational and therapeutic intervention: After an initial introductory session, the second meeting involves generic driving-related skills training. The third involves an analysis of specific traffic violations leading to licence revocations among the group (e.g., driving under the influence). The fourth meeting is designed to encourage drivers to re-evaluate their perceptions of risk in different driving scenarios. This involves recapitulations of the traffic offence (verbalising the sequence of events leading up to the offence, the consequences of the offence, and how it could have been prevented); training in the delivery of first-aid to victims of road traffic accidents; and an activity whereby individual attendees are asked to write an imaginary letter for the real or potential victim(s) of their traffic violation, in which the perpetrator explains the motives behind the offence. The final meeting focuses on the development of personal strategies to avoid risky driving behaviour in the future. To capture any behavioural and/or neurophysiological changes associated specifically with the empathy-induction element of the program, rather than those associated with the generic skills training delivered in weeks two and five, the pre- and post-rehabilitation assessments were conducted, respectively, in the periods between meetings two and three, and four and five. This also allowed us to minimise attrition between the two assessment periods; drop-outs during program occurred most frequently after the initial two sessions. The mean time between assessments was 16.19 (±3.78) days.

Control Drivers had no exposure to the rehabilitation training, and each RD-CD pair met for the first time during the pre-rehabilitation assessment when they were instructed together about the experimental procedure. During each assessment, participants completed the following tasks in the order that they are described below. All the tasks were administered via MATLAB (v2018b; MathWorks Inc.) using the Cogent 2000 toolbox (RRID:SCR_015672; protocol codes and stimuli available at www.osf.io/kv8jm). Participants were recompensed financially upon completion of both the pre- and post-assessment session; CDs received €20 and RDs received €32, given the participation of the latter group in the rehabilitation program.

### 2.3. Campaign videos

To build upon our previous findings [8], first we scanned individuals’ brains while they watched videos designed to elicit empathy and feelings of compassion for victims of road accidents caused by risky driving behaviour. Six videos were selected from the national campaign and nine were chosen from other safe driving campaigns around the world. Each video presented the dramatic depiction of a car accident resulting from dangerous driving (e.g., driving under the influence, speeding). All videos involved 2–3 Caucasian actors of both sexes. As control stimuli, against which brain responses to campaign videos were compared, we selected nine video commercials for different car brands. These adverts were edited to meet the same criteria as the experimental campaign videos. The mean duration of all 24 videos was 27.26 (±3.67; range = 27–31) seconds. Two sequences of stimuli were constructed, each containing a five unique campaign and five of the nine control videos. Both sequences ensured that no more than two campaign or control videos were presented successively. One sequence was presented in the pre-rehabilitation assessment, the other at post-rehabilitation, the order of which was counterbalanced between dyads. To ensure that participants paid attention to the stimuli throughout this Campaign Video (CV) task, at the end of each video they were asked to indicate the number of men or women actors that were involved.

### 2.4. Interactive pattern game

In the interactive Pattern Game (iPG), players build patterns of tokens in either a co-operative or competitive fashion. Developed originally for single-brain imaging [16], we have adapted this game to be an interactive joint-action task for hyperscanning [12]. We used this adaptation to explore whether the rehabilitation program had any effect on brain responses during co-operative or competitive dyadic exchanges.

**Fig 1:**
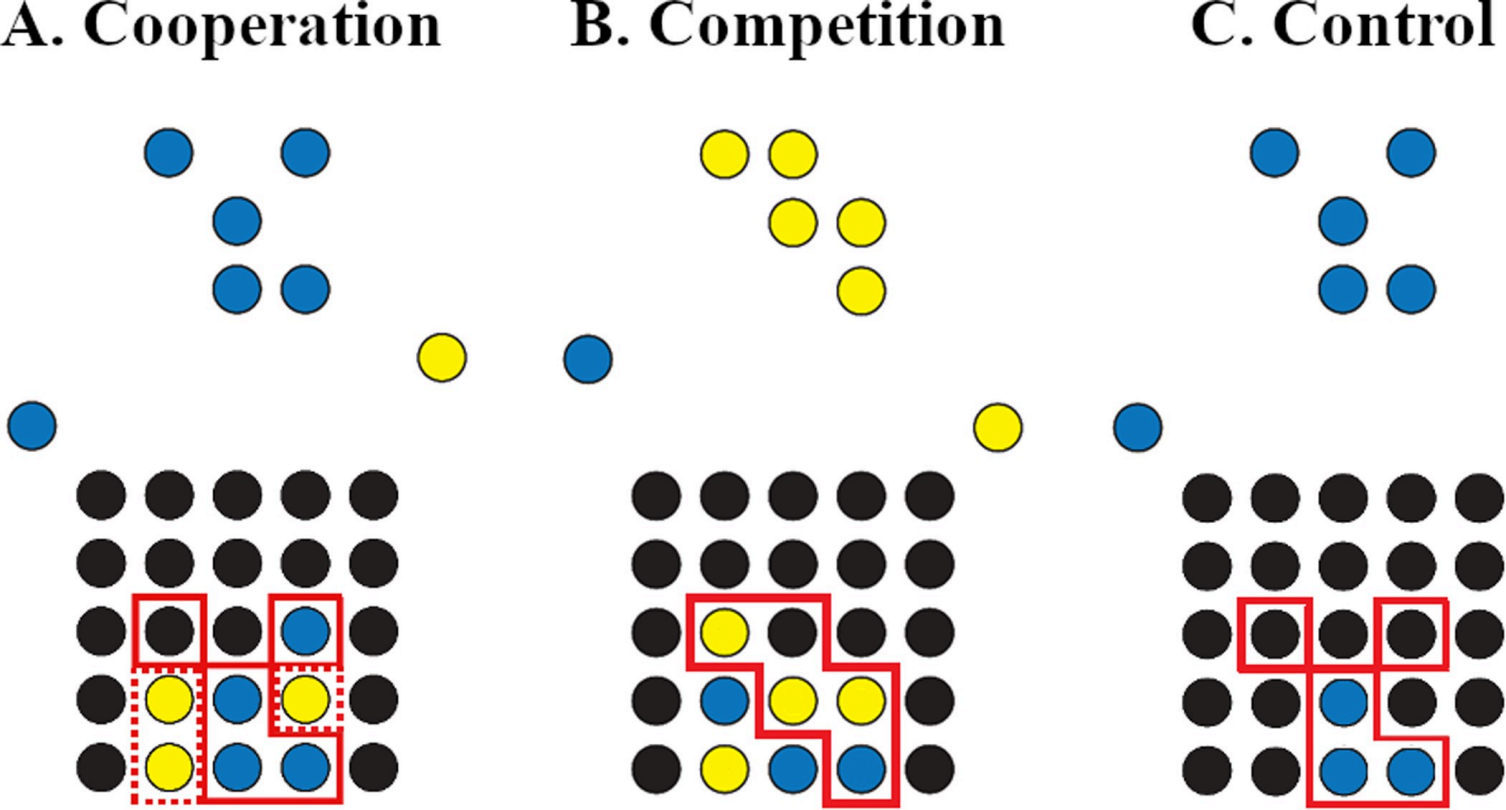
Schematics of the Co-operation (*A*), Competition (*B*) and Control conditions (*C*). In all examples, the Builder is assigned to the same colour as the target pattern and scores by placing tokens in locations that recreate the pattern (solid red lines). In Co-operation and Competition rounds, the Other is assigned to the opposing colour and must place their tokens in locations that serve to help or hinder the Builder (dashed red lines); since the latter is achieved by placing tokens within the pattern space, thereby obstructing the Builder, the scoring location of Others and Builders are the same in Competitive rounds.

A detailed description of the iPG is presented in our previous work [12], and so we describe only the details of its current implementation in this section. Prior to the scanning session, participants were assigned a colour (blue or yellow) that identified them throughout the game and specified the colour of their tokens. At the beginning of each round, they were given an instruction that specified the role for each participant (e.g., “Blue builds, Yellow helps”). The Builder’s aim was to recreate a target pattern of tokens displayed above the playing board. Due to the characteristics of the patterns, the Builder is never able to achieve this perfectly it on their own, and requires assistance from their co-player (the Other). When instructed to help, the Other should place supporting tokens that the Builder needs to assemble the pattern; when told to “hinder” their partner, the Other is required to try to prevent the Builder from reconstructing the pattern by occupying key positions within the playing board. As a Control condition, the Other was instructed to “observe” passively the Builder in recreating the pattern. These instructions defined a Cooperation, Competition and Control condition, respectively. To make the task as interactive as possible, participants placed their tokens simultaneously. The iPG is illustrated in Fig 1.

All pairs played 30 rounds of the iPG (10 Cooperative, 10 Competitive and 10 Control). Player roles changed pseudo-randomly on every round, ensuring that no role was assigned consecutively for more than 3 rounds. Each pattern was composed of five tokens, and each player had five tokens to place on each round. The round ended when the pattern was recreated successfully, when both players had used all their tokens, or when 25 seconds had elapsed.

### 2.5. Driving behaviour questionnaire

After the post-rehabilitation scanning session, participants were asked to complete a computerized version of the Manchester Driving Behaviour Questionnaire (DBQ; [17]) translated into Czech [18]. This self-report instrument comprises 50 items that measure aberrant driving behaviours. On each item, respondents are asked to rate on a six-point Likert scale (1 = “Never”, 6 = “Nearly all the time”) how often they display specific types of driving behaviour. This provides indices of three sub-scales that reflect dissociable types of driving behaviour: “Dangerous violations”, “dangerous errors” and “straying and loss of orientation”. The main distinction between these behaviours involves the degree intention or conscious decision behind an action; while dangerous errors and straying/losses of orientation are characterised by unplanned “slips and lapses” in attention and memory, dangerous violations refer to intentional breaches of traffic rules.

### 2.6. MRI data acquisition

Brain imaging was performed with two identical 3T Siemens Prisma scanners located within the same facility, both equipped with a 64-channel Head/Neck coil. For co-registration, we first acquired high-resolution T1-weightened anatomical images (MPRAGE, TR/TE = 2300/2.33 ms; flip angle 8°; matrix = 240x224x224, 1 mm^3^ voxels). Blood-oxygen-level dependent (BOLD) images were then obtained with T2*-weighted echo planar imaging (EPI), with parallel acquisition (i-PAT GRAPPA accel. Factor = 2; 34 axial slices; TR/TE = 2000/35 ms; flip angle = 60°; field of view = 204 mm × 204 mm; in-plane matrix size = 68 × 68; slice thickness = 4 mm; 34 axial slices; phase encoding = A>P). Axial slices were acquired in interleaved order, each oriented parallel to a line connecting the base of the cerebellum to the base of orbitofrontal cortex to ensure whole-brain coverage. Functional MRI was performed in two separate runs, one for each experimental task: The CV run contained 210 volumes (approx. 7 minutes), and the iPG run comprised 395 volumes (approx. 13 minutes). In both runs, an external programmable signal generator (Siglent SDG1025, www.siglent.com) initiated synchronous acquisition sequences in both scanners, and a single computer was used to present synchronized experimental stimuli to both scanners.

### 2.7. Behavioural data analyses

To measure behavioural performance on the iPG, we recorded the button presses of both players and the ultimate layout of tokens on the playing board. Using this information, we were able to recreate the moves of each player on every round. First, we calculated the number of successful moves a given player achieved in each role and under each condition: For Builders, this was defined as a token placement within a location of the playing board that served to recreate part of the target pattern. The same was true for Hinderers, thereby preventing the Builder from recreating the pattern; while for Helpers, a successful placement was any token that provided support to the Builder. The number of successful moves was then expressed as a proportion of total moves made by the player. These proportions were then entered into a 2 (Group: RD vs. CD) x 2 (Goal: Co-operation vs. Competition) x 2 (Session: Pre- vs. Post-rehabilitation) within-subject ANOVA.

### 2.8. Neuroimaging data analyses

#### 2.8.1. Pre-processing

The pre-processing of structural and functional brain images was performed using various utilities within FMRIB’s software library (FSL; [19]; SCR_002823). Across the pre- and post-rehabilitation assessment sessions, for each participant we acquired two anatomical scans and four functional time-series (2 tasks x 2 sessions) that were pre-processed individually. Anatomical images were skull-stripped using BET [20]. Using FEAT [21], slice-timing correction for interleaved acquisition was applied to the functional images, and each time-series was high-pass filtered across time (Gaussian-weighted least-squares straight-line fitting; sigma = 50.0 secs) and spatially smoothed with a 5mm full-width half-maximum Gaussian kernel. Motion correction was competed by MCFLIRT [22]. We then performed Independent Component Analysis (ICA) with MELODIC [23] to identify artefactual signals reflecting residual head motion or physiological noise (e.g., heartbeat, respiration). This analysis performed an extraction of 50 spatio-temporal components of the BOLD signal, from which we identified artefactual signals automatically with the Spatially Organized Component Klassifikator (SOCK; [24]. Signals expressing the artefactual components were then regressed out of the time-series using the *fsl_regfilt* utility. Finally, the pre-processed time-series were registered to their corresponding high-resolution anatomical image using boundary-based registration in FLIRT, and this, in turn, was registered linearly to the MNI-152 template.

#### 2.8.2. General linear modelling

With FEAT, we applied General Linear Modelling (GLM) to the 108 functional time-series (2 sessions x 54 drivers [27 pairs]) acquired during each experimental task separately to investigate whether fMRI could detect changes in brain responses between pre- and post-rehabilitation expressed in RDs but not CDs. We also assessed whether the findings of our previous study could be reproduced in this independent sample; specifically, whether RDs exhibited a reduced brain response to campaign videos compared with CDs before the rehabilitation program.

For the CV task, fixed-effect analyses were first performed on individual time-series to determine parameter estimates for the contrast campaign > control videos. The resulting parametric maps were then carried forward for group-level whole-brain random-effects analyses with FLAME; specifically, an independent-sample t-test that compared RDs and CDs at pre-rehabilitation, and then a 2 (Session: Pre vs. Post) x 2 (Group: RD vs. CD) analysis of variance (ANOVA) that assessed changes after rehabilitation that were specific to RDs.

For the iPG task, we used GLM to identify brain signals elicited as a reaction to the co-operative or competitive behaviour of an interaction partner. We refer to this as interpersonal brain-behaviour coupling. Using an approach that we developed previously [12], in an event-related fashion we modelled the brain response of each individual in the 1-second period immediately following each of their partner’s token placement. We then assessed whether these reactive brain responses changed between the pre- and post-rehabilitation assessment in RDs but not in CDs. Using the same two-step GLM process, fixed-effect analyses were first performed for the following parameter estimations at the individual level: Builders’ brain responses to the moves of the Other under the Co-operation (COOP) or Competition condition (COMP), and while attempting to recreate the pattern without any help or hindrance (CTRL). Importantly, by modelling brain responses recorded during a player’s own token placement in the Control condition we were able to distinguish between those reflecting a reaction to their partner’s token placement and those elicited during their own subsequent action (see below). Group-level whole-brain random-effects analyses of variance were then performed in FLAME; specifically, 2 (Session: Pre vs. Post) x 2 (Group: RD vs. CD) ANOVAs were applied to the contrast [COOP>CTRL] *vs*. [COMP>CTLR] to determine whether reactive brain responses elicited specifically during co-operative or competitive exchanges differed between the pre- and post-rehabilitation assessment in RDs but not CDs.

Since non-parametric permutation inference offers more precise control over false positives than other methods of multiple-comparison correction [25], group-level statistical maps were corrected across space using the FSL utility *randomise* [26] with 5000 permutations and threshold-free cluster enhancement [27].

#### 2.8.3. Inter-subject correlation

To assess whether inter-brain alignment could be observed between interacting RD-CD pairs during co-operative and/or competitive exchanges in the iPG, and if expressions of alignment changed after the rehabilitation program, we performed a data-driven analysis of inter-subject correlation (ISC). A detailed description of this technique is presented elsewhere [13], so only the details of its current implementation is described in the following section.

First, we applied principle component analysis (PCA) to each of the 108 time-series to identify spatially orthogonal patterns of brain response, the number of which was determined by the minimum description length [28]. Any pattern, or component, expressing spatial or temporal characteristic that resembled residual head motion or physiological noise (e.g., respiration, heartbeat, cerebrospinal fluid) were omitted from further analyses. Using the GIFT toolbox for MATLAB (v2.0e; mialab.mrn.org/software/gift; [29]), group independent component analysis (gICA) was then performed 20 times on the non-artefactual components using the INFOMAX algorithm to identify those that were expressed reliably and independently of one another at the group level. From the most reliable components, we then identified those that were expressed in individuals’ brains along a time-series that corresponded to the onsets and durations of co-operative or competitive exchanges. Using the results of the PCA, each non-artefactual component was then back-reconstructed to each of the 108 input time-series to produce a subject-specific time-course for that pattern in each assessment. Multiple regression analyses were then computed to identify components that were expressed specificity across either COOP or COMP rounds in *both* assessments: For each subject, the explanatory variables were their back-reconstructed time-course for each independent component and the outcome variable was their unique task design for either the COOP, COMP or CTRL condition. This resulted in three subject-specific *β*-values for each component, and Bonferroni-corrected paired-samples *t*-tests were conducted to identify task-specific components (*β*_COOP_ > *β*_CTRL_ and *β*_COMP_ > *β*_CTRL_).

Finally, to examine whether the time-series of BOLD signals covaried between interacting players during co-operative and/or competitive exchanges, for each interacting pair we computed Pearson cross-correlations between the back-reconstructed time-series for components expressing task specificity. The resulting correlation coefficients were transformed to *z*‐values, and the median was used as a coefficient of alignment. To determine the significance of the resulting coefficient, we performed a randomization test with 10,000 permutations: in each iteration, we randomly selected pairs among the 53 non-interacting players and computed a median z‐transformed coefficient as above. This produced a null distribution of correlations among non-interacting pairs, against which the significance of alignment between each interacting pair was then compared. Finally, to assess whether the degree of inter-subject correlation changed after rehabilitation, we compared interaction-specific coefficients of alignment among between the pre- and post-rehabilitation.

## 3. Results

### 3.1. Behaviour

#### 3.1.1. Interactive pattern game

The ANOVA conducted on the proportion of successful token placements achieved by each participant on the iPG revealed a main effect of Goal; all players made more successful placements the Co-operation relative to the Competition condition (.87 [±.02] vs .42 [±.01]; F[1,26] = 368.92, p < .001, ηp^2^ = 0.93). A main effect of Group demonstrated a higher proportion of successful placements achieved by CDs compared with RDs (.68 [±.02] vs .60 [±.02]; F[1,26] = 15.63, p = .001, ηp^2^ = .38). The main effect of Session showed that participants were more successful in the Post- relative to the Pre-program session (.66 [±.01] vs .63 [±.02] ms; F[1,26] = 9.80, p = .004, ηp^2^ = 0.27). Interestingly, a significant Goal-by-Group interaction (F[1,26] = 17.04, p < .001, ηp2 = .396) revealed that, while CDs and RDs were similarly successful in the Co-operation condition (.87 [± .02] vs .86 [±.03]), CDs made more hindering token placements compared with RDs in the Competition condition (.49 [± .02] vs .35 [±.02]). A Goal-by-Session interaction (F[1,26] = 6.01, p < .02, ηp^2^ = .19) revealed an increased proportion of successful token placements in the Post- relative to the Pre-rehabilitation assessment session under the Co-operation (.84 [± .03] vs .89 [± .02]) but not the Competition condition (.41 [± .01] vs .42 [± .01]). Crucially, however, there was no significant Group-by-Session interaction (F[1,26] = .11, p = .470, ηp^2^ < .01), nor a three-way Goal-by-Group-by-Session interaction (F[1,26]<0.01, p = .960, ηp^2^ < .01).

#### 3.1.2. Driving behaviour questionnaire

To assess differences in self-reported driving behaviour between RDs and CDs, we compared the groups on items of the DBQ measuring the errors and violations sub-scales. Since responses on the instrument did not meet the assumption of normality, comparisons were performed with non-parametric Mann-Whitney tests. Higher ratings across items measuring dangerous errors were reported by CDs compared to RDs (medians = 16 vs 14; ranges = 11–21 vs 10–22; U[27] = 244, p = .018), but those across items measuring violations were marginally higher in RDs compared to CDs (medians = 26 vs 24; ranges = 17–44 vs 17–42; U[27] = 269, p = .049). There was no difference between RDs and CDs in responses to items measuring straying and loss of orientation (medians = 33 vs 35; ranges = 23–48 vs 25–50; U[27] = 306, p = .158).

### 3.2. Neuroimaging

#### 3.2.1. Campaign videos

**Fig 2:**
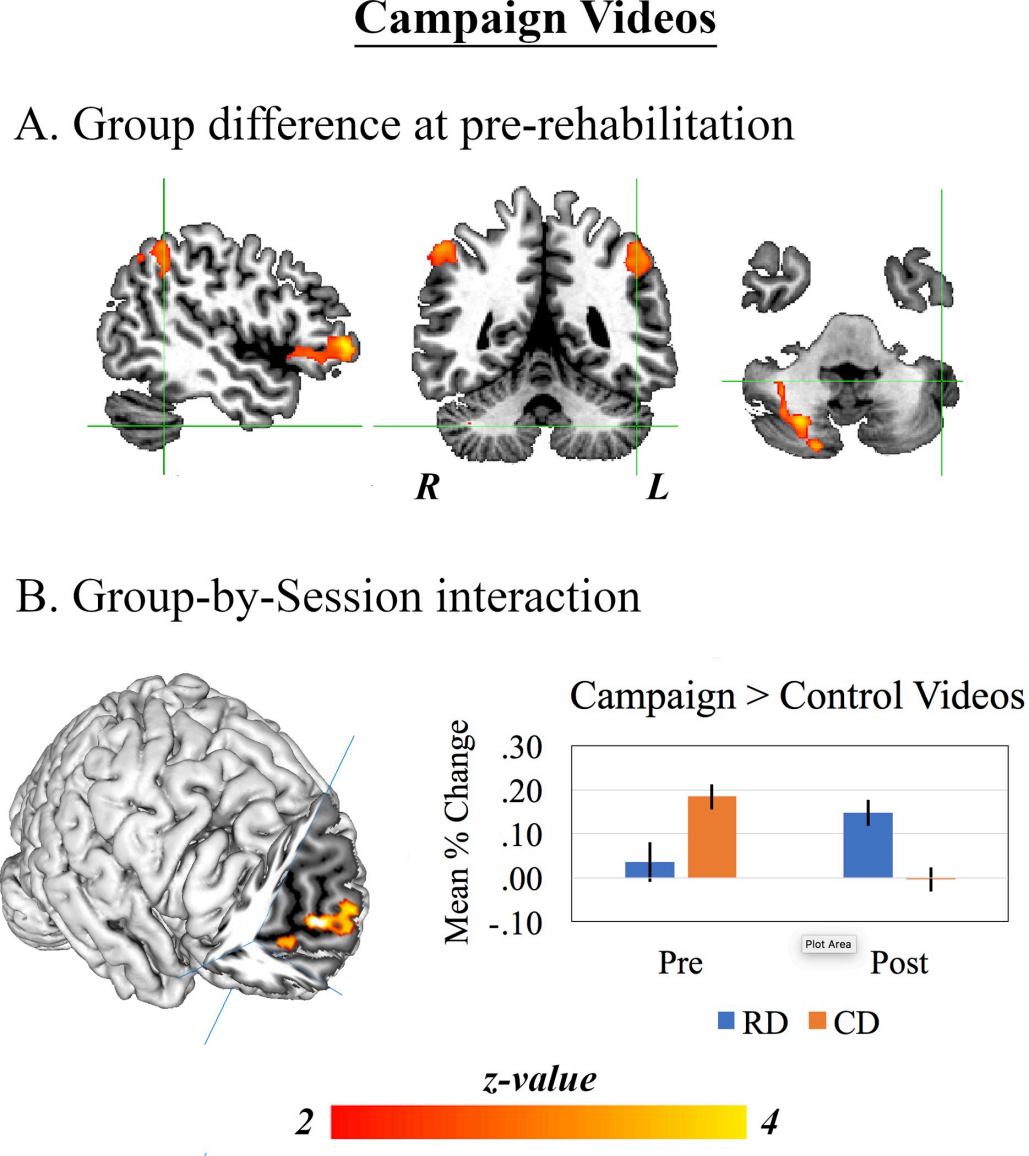
Results of the GLM analyses applied to data acquired during viewing of the campaign videos. *A*: Clusters of brain response in which the campaign>control video contrast was expressed significantly more in CDs than RDs during the pre-rehabilitation assessment. *B*: The single cluster of brain response within the medial prefrontal cortex in which the campaign>control contrast expressed an increase in RDs but a decrease in CDs between pre- and post-rehabilitation. The mean percent signal change extracted from all voxels comprising this cluster are also presented. *Note*: All whole-brain z-maps are shown after non-parametric permutation inference involving 5000 permutations, thresholded at *p* < .05.

Within both the RD and CD groups, drivers correctly recalled the number of male or female actors in over 80% of videos. A comparison of BOLD signals expressing the campaign > control videos contrast between RDs and CDs revealed a greater relative increase in the latter group within the left inferior frontal gyrus, extending into the anterior insula; bilateral posterior and inferior parietal cortex, encompassing the intraparietal sulci; and the right cerebellum. This contrast also revealed a Group-by-Session interaction: the greater relative increase RDs exhibited a significant increase in their neural response to the campaign videos between pre- and post-rehabilitation, while SDs showed a significant decrease. This interaction was expressed by a cluster of BOLD response localised primarily to the medial prefrontal cortex (mPFC) and adjacent anterior cingulate cortex (ACC), but also within the right cerebellum. These results are illustrated in Fig 2, and details are provided in Table 1.

**Table 1:** Clusters expressing group and Group-by-Session effects for contrasts of interest.

		Label	Voxels	Peak z	X	Y	Z
	CD_Pre_ > RD_Pre_						
CV Task	L	Cerebellum	500	3.94	26	-70	-40
	L	L IPL	346	3.39	-56	-52	40
	L	L IFG	314	4.27	-48	44	0
	R	R IPL	185	3.44	56	-50	50
	R	R PPC	52	3.11	42	-64	48
	RD_Post>Pre_ > CD_Post>Pre_						
	L	mPFC	669	5.4	-8	54	20
	R	Cerebellum	5	4.77	16	-62	-46
iPG Task	RD_Post>Pre_ > CD_Post>Pre_						
	R	mPFC		2.58	4	64	20
	L	ACC		2.66	-2	44	14
	RD_>Pre_ > CD_Pre_						
		ACC	358	3.34	0	44	14

Greyed rows indicate a cluster expressing the Group-by-Session effect only at an uncorrected (p < .01) level.

### 3.3. Inter-personal brain-behaviour coupling

**Fig 3:**
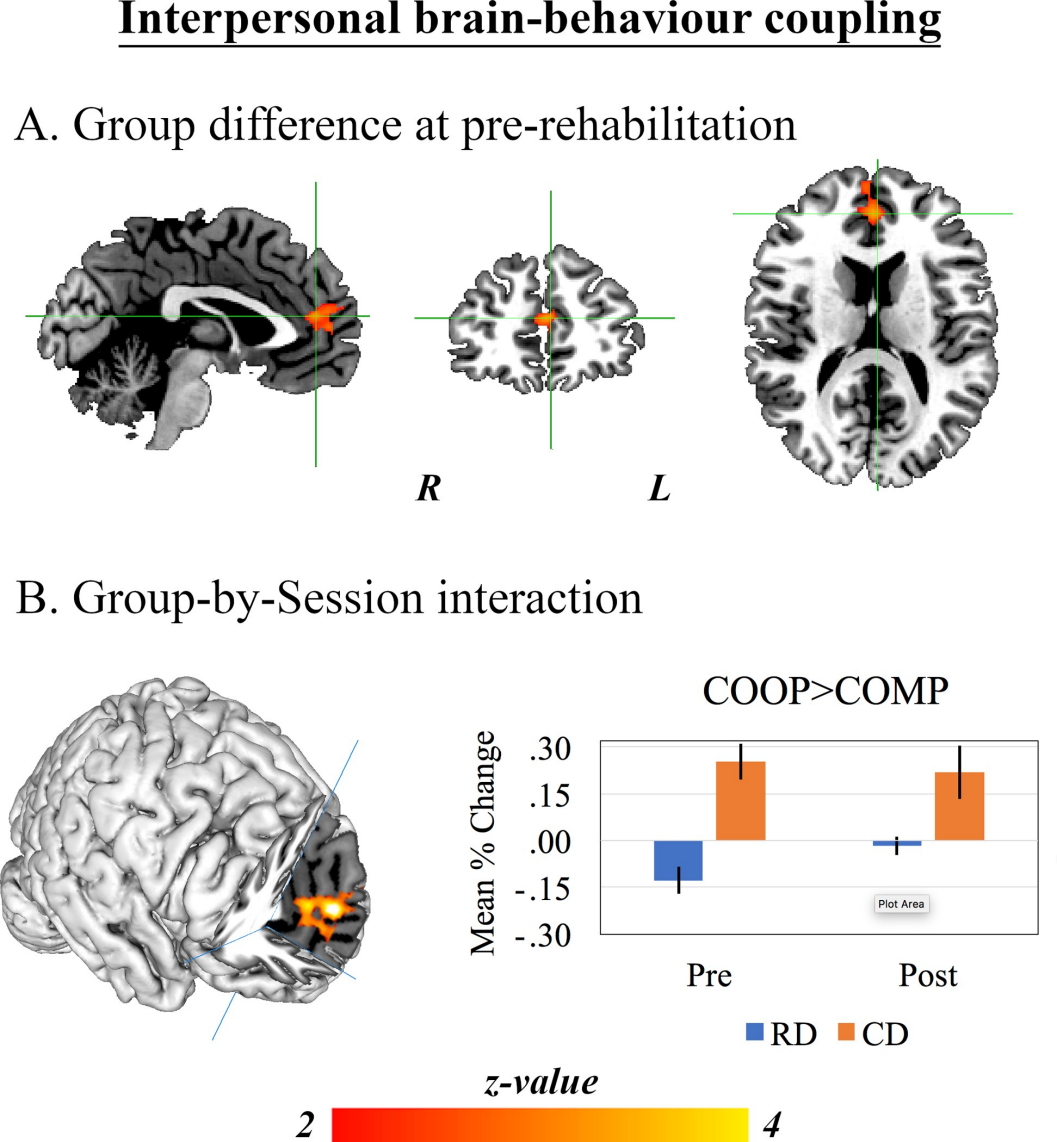
Results of the GLM analyses applied to data acquired during the interactive pattern game. *A*: The single cluster representing neural reactions to the behaviour of a co-player during pre-rehabilitation that were stronger in the COMP>CTRL contrast for RDs but the COOP>CTRL contrast in CDs. The whole-brain z-map is presented after non-parametric permutation inference involving 5000 permutations, thresholded at *p* < .05. *B*: The single cluster of brain response within the medial prefrontal cortex in which the [COOP>CTRL] *vs*. [COMP>CTRL] contrast expressed a Group-by-Session interaction at the uncorrected level (*p* < .01), but did not survive non-parametric correction for multiple comparisons. This is presented only to illustrate our motivation for session-specific assessments of the Group effect, which revealed differences in and around this functional cluster at pre- but not post-program assessment after multiple-comparison correction (see text).

After non-parametric correction, the pre-defined Group-by-Session interaction was not observed in any BOLD signal expressing either the [COOP>CTRL] *vs*. [COMP>CTLR] contrast. Given the results of the former analyses, however, it is noteworthy that the uncorrected (p < .01) group-level maps suggested a weak session-specific difference might exist between the groups; in a single cluster of BOLD signal expressed within the mPFC, RDs exhibited greater reactive responses to the behaviour of their co-player during COMP relative to COOP rounds before but not after rehabilitation, while CDs exhibited equally greater reactive brain signals in the COOP compared with the COMP condition in both assessment sessions. To investigate this further, we assessed the Group effect in the [COOP>CTRL] *vs*. [COMP>CTR] contrast in pre- and post-program assessment separately. Indeed, a cluster of BOLD signal within the mPFC and adjacent ACC expressed the Group effect during the pre- but not the post-program assessment after non-parametric correction. This is illustrated in Fig 3.

### 3.4. Inter-subject alignment

**Fig 4:**
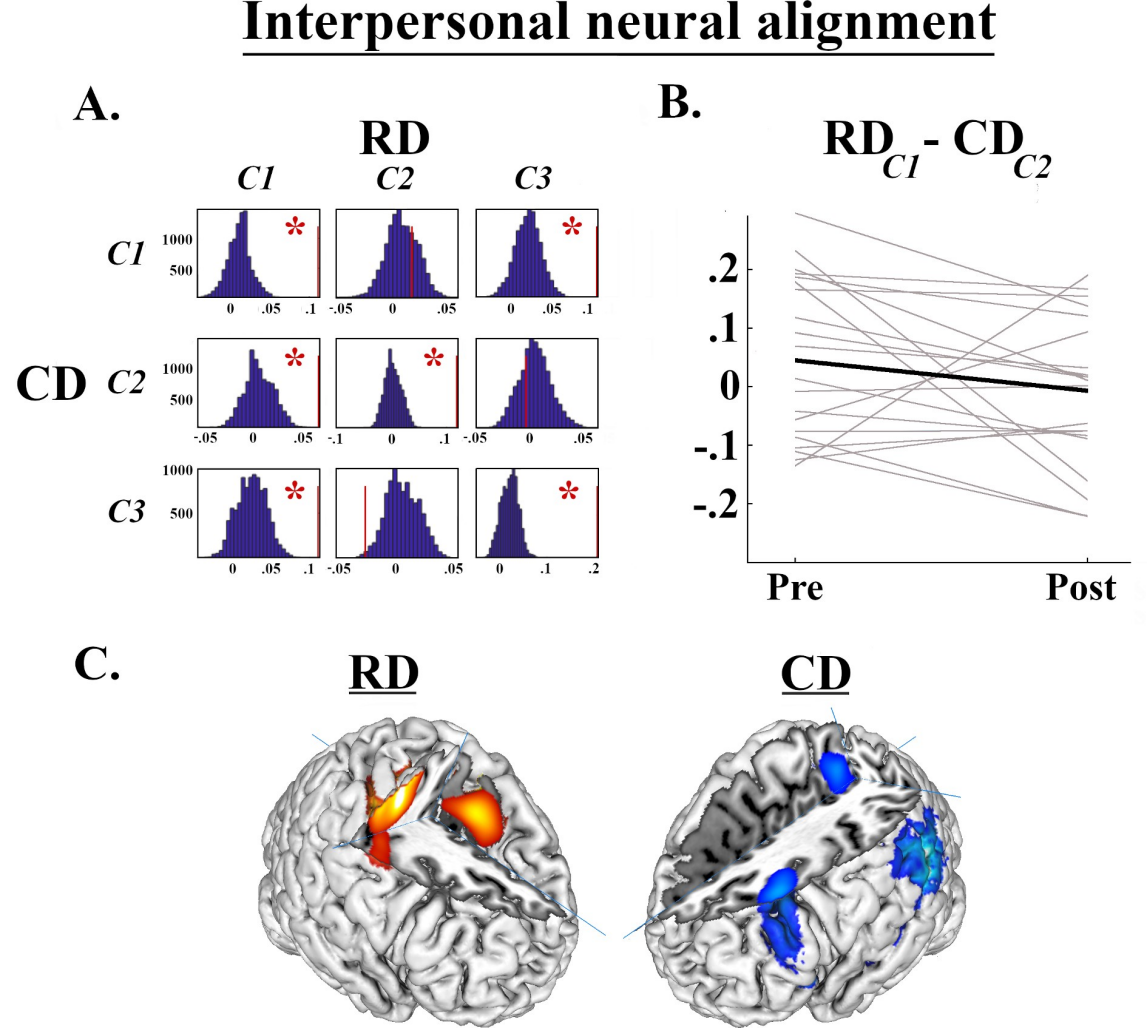
Results of inter-subject correlations. *A*: Median coefficients of alignment among interacting pairs within components expressing task specificity (red lines), plotted against the null distribution of coefficients among non-interacting pairs (blue histograms). Red asterisks indicate alignment between components that was significantly greater among interacting than non-interacting pairs (e.g., C1 in RDs and C2 in CDs [RD_*C1*_-CD_*C2*_]). *B*: Alignment coefficients between RD_*C1*_ and CD_*C2*_ for each pair at pre- and post-rehabilitation assessment. *C*: The spatial distribution of neural signals comprising C1 and C2.

The initial PCAs identified a set of 17 spatially orthogonal, non-artefactual principal components, which were then fed into 20 iterations of gICA to assess their reliability. This confirmed that all 17 were expressed reliably across the sample (cluster-quality indices >.97). Of these components, only three had back-reconstructed time-series that demonstrated any significant task-specificity in *both* the pre- and post-rehabilitation assessment; namely, one component expressed specifically during competitive rounds (*β*_COMP_ > *β*_CTRL_; C1) and two expressed on both competitive and co-operative exchanges more than control rounds (*β*_COOP_ > *β*_CTRL_ and *β*_COMP_ > *β*_CTRL_; C2 and C3). As shown in Fig 4A, Bonferroni-corrected comparisons of the inter-subject correlation among interacting and non-interacting pairs identified six combinations of components in which significant alignment was observed. In only one of these combinations did alignment differ significantly between the assessment sessions, however; specifically, alignment decreased between C1 expressed by RDs and C2 in CDs (p_corr_ = .037; see Fig 4B). Component C1 encompassed the dorsal extent of bilateral pre- and post-central gyri, the supplementary motor area, and bilateral clusters within the cerebellum. Component C2 was expressed in bilateral superior temporal cortex, extending into the temporo-parietal junction on right side; right superior middle frontal gyrus and the precuneus. This is illustrated in Fig 4C.

## 4. Discussion

By utilizing some of the most recent advancements in function magnetic resonance imaging (fMRI), this study performed a neuroscientific evaluation of a driver rehabilitation program that employs empathy induction as a means of changing attitudes towards risky driving behaviour. By scanning the brains of drivers who had lost their license through serious motoring offences (RDs) before and after their involvement in the empathy induction element of the program, we assessed the effectiveness of this rehabilitation technique at the neurophysiological level. Three primary findings emerged, which together demonstrate not only the effectiveness of empathy induction for driver intervention but also the value of fMRI for the evaluation of rehabilitation programs.

First, by comparing brain responses to empathy-eliciting stimuli between RDs and the group of control drivers who did not engage in the rehabilitation program (CDs), we demonstrate a selective increase in the former group after their participation in the rehabilitation program. This pattern of change in brain responses was localized to the medial surface of the prefrontal cortex (mPFC), extending into the anterior cingulate cortex (ACC). The mPFC is engaged consistently by tasks that require individuals to infer the beliefs, mental and intentional states of others [30,31]. Similarly, the ACC is involved in empathy for others’ pain [32]. This suggests that the rehabilitation program is capable of enhancing the socio-emotional processing that is otherwise blunted in these drivers. At first glance, it might seem surprising that such a short period of socio-affective training is sufficient to induce changes in empathy-related neurophysiological responses. This is not the first study to observe such effects, however: Empathic expression is subject to top-down influences, and even a simple instruction to behave more empathetically is sufficient to elevate empathy-related brain responses within the mPFC [33]. Unsurprisingly, then, a short period of structured training is reported to be effective at enhancing neural indices of empathic expression; Klimecki et al. [34], for example, report that 6 hours of empathy training delivered in a single day was sufficient to increase neural responses to videos depicting human suffering within brain regions associated with empathy for pain (e.g., dorsal ACC). Similarly, the same amount of compassion training has been shown to elevate brain responses during the observation of human suffering [35], and four weeks of daily compassion meditation training is reported to increase charitable donations [36]. It has been suggested that this reflects the top–down cognitive modulation of empathic responses–empathy training appears to increase an individual’s tendency or willingness to intentionally empathise [36].

It remains unknown how long the neural and behaviour effects of social-emotional training last. Despite evidence that empathy training can induce effects lasting up to six months [37], these effects are measured most frequently with behavioural and self-report assessments that can provide unreliable estimates of efficacy [38]. This is true especially for driving offenders; it is likely that the responses of these individuals will be prone to social desirability if they believe certain answers will help them retrieve their license. This makes it difficult to assess whether an intervention was effective in modifying the target attitude or behaviour in the long term. Our data demonstrate the potential for neuroimaging techniques to overcome these challenges, providing a more objective and reliable measurement. Future research should consider building on our findings to determine how long the changes between pre- and post-rehabilitation last.

It is suggested that empathy likely plays a role in the degree to which individuals engage in prosocial and antisocial behavior [39]. Consistent with this notion, research suggests that people who show greater physiological empathic responses during the observation of another’s pain are more likely to make decisions that prevent others from feeling pain in the future, even at the cost of their own comfort [40]. In this light, an individual’s empathic awareness, or their *willingness* to express empathy for others, might have the potential to influence their driving behaviour. Since the sample of RDs assessed in this study were unwilling to provide follow-up data after their involvement in the experiment, we are unable to evaluate this directly. Interestingly, the elevated brain responses we observed within the mPFC of these drivers after empathy induction was not accompanied by a change in their performance on either co-operative or competitive exchanges of the interactive Pattern Game. This might indicate that any changes to an individual’s willingness to empathise brought about through empathy induction might not translate directly into a higher propensity for prosocial behaviour. Alternatively, this finding might reflect the inadequacy of performance on this experimental task as an index of real-world behaviour. In a similar vein, the relatively small difference in self-reported traffic violations between drivers with no formal motoring convictions and those who had lost their licences because of one or more serious traffic offences suggests that such subjective measures are insufficient to capture real-world driving behaviour. Future studies should build on our findings to assess the degree to which changes in responses following empathy induction are associated with actual driving behaviour, such as an individual’s propensity to retaliate against the perceived wrong-doings of other road users.

This brings us to our second finding: by performing fMRI on pairs of drivers whilst they interacted with one another, data acquired at pre-rehabilitation reveal significant differences between the driver groups in their neural reactions to a co-player’s behaviour; at pre-rehabilitation, this interpersonal brain-behaviour coupling was greater during competitive exchanges in RDs but co-operative exchanges for CDs. This difference was again observed within the mPFC and adjacent ACC. Furthermore, although the group-by-session interaction was not significant, this difference was no longer present at post-rehabilitation due to a selective change in RDs. Other studies have reported that risky drivers exhibit reduced inhibitory control and a correspondingly attenuated neurophysiological correlate (P3 component of the event-related response) when confronted with emotionally salient stimuli relative to other individuals [41]. The greater reactive brain responses during competitive relative to co-operative exchanges that we have observed in our sample of RDs *before* rehabilitation might reflect their greater proclivity to react to negative environmental cues in the face of diminished inhibitory regulation. This may well lead to risky driving behaviour. Our paradigm permits novel insights into this disinhibited aspect of driver behaviour; by moving away from third-person neuroimaging, in which individuals’ brains are scanned while they observe stimuli passively, and employing a second-person approach whereby brains are measured during bidirectional and reciprocal exchanges, we have shown that the reactive brain responses of RDs can be modulated by rehabilitation that incorporates empathy induction. As an extension of our findings, it would be useful to see whether these changes in interpersonal brain-behaviour coupling following rehabilitation relate to different elements of driving behaviour.

Finally, with dual-fMRI hyperscanning we show stronger inter-brain alignment between interacting RDs and CDs at pre- relative to post-rehabilitation; specifically, the signals measured within discrete neural motor circuits in RDs and among brain systems implicated in socio-cognitive processes in CDs become less correlated after rehabilitation. At first glance this appears to be counter-intuitive; numerous studies report between-brain alignment during co-operative interactions, attributing such synchronization to the establishment of shared meaning or the successful transmission of information (e.g., [42,43]. We suggest this decrease from pre- to post-rehabilitation reflects a change in the neural systems engaged in each session. Prior to rehabilitation, alignment was observed in the motor circuits of RDs’ brains. Together with the reduced neural responses to empathy-eliciting stimuli and the greater interpersonal brain-behaviour coupling during competitive exchanges seen in RDs during the initial assessment, alignment within motoric brain systems might reflect their tendency to react to their partner’s behaviour without consideration of their underlying motives or intentions. In contrast, the distributed set of brain regions in which neural signals align in CDs comprise the dorso-lateral prefrontal cortex, posterior superior temporal cortex and temporo-parietal junction, and the precuneus–regions comprising the inter-connected “mentalising” network, through which we generate inferences about the beliefs of others and their mental states [44,45]. Future studies might attempt to evaluate this interpretation by comparing between-alignment within dyads comprising individuals who have *both* engaged in the rehabilitation against dyads consisting of two interactants who have not engaged in the program.

Our study is certainly not the first to employ fMRI in the context of driving-related behaviours (e.g., [9,46,47], road-traffic accidents (e.g., [48] or traffic safety (e.g., [8,49]. To our knowledge, however, this is the first use of functional neuroimaging to evaluate driver-rehabilitation programs. Taken together, our intra- and inter-personal analyses of fMRI data demonstrate the effectiveness of a rehabilitation program that utilizes empathy induction as a means of attitudinal change. With a hyperscanning paradigm sensitive enough to detect changes in empathy-related brain responses, interpersonal brain-behaviour coupling and neural alignment between pre- and post-rehabilitation, we hope that future research develops our methods in an attempt to improve existing rehabilitation programs or develop new ones.
